# Notes on the Decay of Human Teeth

**Published:** 1887-01

**Authors:** W. D. Miller

**Affiliations:** Berlin.


ARTICLE II.

### NOTES ON THE DECAY OF HUMAN TEETH.

BY PROF. W. D. MILLER, BERLIN.

Four or five years have now elapsed since what may be called the chemico-parasitic theory of tooth decay came into prominence, and since that time it has been very steadily gaining ground. A very great obstacle in the way of its advancement has been the general lack of information as to the conditions of growth and physiological action of fungi, particularly those of the human mouth. It is, however, to be hoped that the work begun by the Illinois State Dental Society, under the leadership of Dr. Black, will be taken up by other societies, and soon bring about a better understanding upon this most important subject.

It is not my object in this paper to go over the ground which I have pretty thoroughly traversed in other papers, but rather to call attention to some isolated points in the etiology of decay, as well as to a few points where I have not been quite correctly interpreted.

While many of the views regarding dental decay which

I labored to establish two or three years ago are now accepted without reserve, and others have lately been completely confirmed by Drs. Black, Sudduth and others, I am not aware that a single point has been made by anyone which could in any way impair the validity of the conclusions at which I then arrived.

*On the Physiological Action of Fungi.*—There seems to be not a little misunderstanding, even among those who have given more or less attention to the subject, as to the physiological or chemical action of the fungi of decay, and the opinion is prevalent that during the first stage (decalcification) one fungus is present, but during the second (solution of the softened matrix) another. Arkovy even goes so far as to assume a special organism for *Caries chronica*, another for *Caries acuta*, and a third for *Caries acutissima*, &c.

I have already clearly demonstrated in this journal, that any fungus of the human mouth, whether temporary or permanent, which can affect a fermentation of starch or sugar, may be instrumental in bringing about the first stage of decay; that any which possesses a peptonizing action may, by dissolving the softened dentine, produce the second stage; and that any which possesses both properties (and there are many such in the human mouth) may accomplish the whole process of decay. The micro-organism which produces the decalcification may also produce the solution of the decalcified substance.

I have also shown in these pages that the reaction produced by a given fungus depends, in many cases at least, upon the nature of the culture medium. For example, the comma bacillus which I found in the human mouth liquefies the boiled white of egg (it also liquefies decalcified dentine), with the development of strongly alkaline products and offensive odors; in beef extract sugar solution the reaction is distinctly acid, with no trace of bad smelling products. The reaction of a solution containing a pure culture of a fungus can, in the majority of cases, be made neutral, alkaline or acid, at will, by varying the relative amounts of albuminous and saccharine substances present in the solution. In a like manner the reaction in a cavity of decay must depend to some, if we may not say to a great extent, upon the relative amounts of nitrogenous and non-nitrogenous materials in the cavity. This fact will explain an appearance frequently to be met with in the oral cavity. We find a tooth badly broken down, the pulp ulcerated or gangrenous, the gum, having grown into the cavicy and constantly irritated by the sharp edges of the tooth, likewise inflamed and suppurating We have here an excess of nitrogenous material, and a putrid, alkaline condition. Instead of a thick layer of softened dentine, we find a thin, black, or dark brown layer of comparatively hard dentine, a condition which has led to the statement that decay of pulpless teeth is essentially different from that of normal teeth.

We need not go far for an explanation. The already softened dentine has been for the most part dissolved, and, owing to the *present* alkaline condition, no farther decalcification can take place. From this condition to one of rapidly advancing decalcification we find every transition.

*Decay of Bakers' Teeth.*—One of the most convincing features in favor of the chemico-parasitic theory is its ability to account for the most diversified phenomena of decay. A striking proof of this is furnished by an article on the above subject from Prof. Hesse, in the *Deutsche Monatsschrift.** Hesse finds that bakers suffer to a surprising extent from decay of teeth, affecting principally the labial surfaces. He attributes it to the fact that bakers constantly breathe in flour, which is deposited upon the surfaces of the teeth, where it speedily ferments, after being converted into sugar by the diastase of the saliva. I recorded in this journal an experiment in which a glass tube filled with flour and tied to a tooth in the mouth showed a strong acid reaction in a few hours. Hesse looks upon the rapid decay of bakers'

* A translation of this article will be found in the September number of the *Independent Practitioner.*

teeth as a confirmation of the theory which I have supported.

*Caries under Fillings.*—In regard to this subject, I have not been quite correctly understood. *All* bacteria require moisture for their proliferation. The majority of them (the ærobes) require oxygen ; a few (the anærobes) grow better without oxygen ; some grow equally well with or without (here belong a number which I have met with in the mouth) while very many, if not all, may subsist for a short time on the oxygen contained in the medium in which they are found.

From these facts every one may draw his own conclusions. If softened dentine is left in a cavity it should, in every case, be perfectly sterilized and dried (with warm air) before filling, and the filling must, of course, be water tight. Only under these conditions can we with certainty prevent the softened dentine from farther decay, since the mere exclusion of air is no guarantee against the action of the fungi.

*Lime-Salts as Antacids.*—The lime-salts of the tooth are usually spoken of as antacid, and therefore are speedily neutralizing the acids of decay. This is only in part right. The carbonite of lime is antacid, but the phosphate, which makes up the great bulk of the lime-salts, is not, *i. e.*, it cannot neutralize the acids of decay. Add as much phosphate of lime to a weak solution of lactic acid as it will dissolve, or even an excess, and it will be found to be as strongly acid as before, and in this condition it still appears to retain the power of softening dentine, though not as rapidly as an equally strong solution to which no phosphate has been added.

A drop of lactic acid applied to dentine does not, therefore, extract that amount of lime-salt which is necessary to neutralize it, but rather that which is required to form a saturated solution of the phosphate after, of course, deducting the amount which has been neutralized by the carbonate. In another paper I will discuss this point more fully, and endeavor to present some interesting facts which grow out of it.

*Test for Lactic Acid in Decaying Dentine.*—Prepare a mixture of carbolic acid and chloride of iron, as described in previous numbers of this journal (the color must not be too deep). Put about 3 c c., say a large thimbleful, in a test-tube, and carefully add the softened dentine from a decaying tooth; let it stand from ten minutes to two hours in a dark place, and a yellow color will usually appear around the pieces of dentine, indicating lactic, citric, tartaric or malic acid, and in consideration of the fact that lactic acid fermentation has been proved to be constantly going on at certain points in most human mouths, furnishing pretty conclusive proof of the presence of the first mentioned acid in decaying dentine.

This is the same result which I arrived at by the very laborious and difficult process of treating large quantities of decayed dentine with sulphuric acid, extracting with ether and forming the zinc-salt. The amount of acid present in a decaying tooth is not always sufficient to produce the reaction clearly.—*Independent Practitioner.*

